# A Call to American Physicians, from the Red Cross

**Published:** 1918-09

**Authors:** 

**Affiliations:** American Red Cross, Washington, D. C.


### Abstracts and Selections.

### To Physicians of America.

Surgeon General Gorgas has called for 1,000 graduate nurses a week—8,000 by October 1.

Twenty-five thousand graduate nurses must be in war service by January 1—in the Army Nurse Corps, in the Navy Nurse Corps, in the U. S. Public Health Services, in Red Cross war nursing.

This involves withdrawal of many nurses from civiilian practice and necessitates strict economy in the use of all who remain in the communities.

You can help get these nurses for our sick and wounded men by:

Bringing this need to the attention of nurses.

Relieving nurses where possible wholly or in part from office duty.

Seeing to it that nurses are employed only in cases requiring skilled attendance.

Insisting that nurses be released as soon as need for their professional service is ended.

Seeing that your patients use hospitals instead of monopolizing the entire time of a single nurse.

Encouraging people to employ public health nurses.

Instructing women in the care of the sick.

Inducing high school and college graduates to enter the Army School of Nursing or some other recognized training school for nurses.

Encouraging nurses to go to the front involves real personal sacrifice and added work on the part of the physicians whose duty it is to maintain the health of our civilian second line defense.

But the men who are fighting for their country in France need the nurses.

Department of Nursing,
American Red Cross,
Washington, D. C.

